# *Rosa roxburghii* Fermentation Broths Attenuate Bleomycin‐Induced Pulmonary Fibrosis by Activating the Nrf2/HO‐1/NQO1 Signaling Pathway and Modulating Gut Microbiota

**DOI:** 10.1002/fsn3.70105

**Published:** 2025-03-19

**Authors:** Heting Zhou, Xinyue Zheng, Shaolin Huang, Xiaomeng Wang, Ting Zhou, Shuwen Zhang, Yihan Ling, Wenxi Wang, Xingjie Li, Shouqian Li, Yongmei Xie, Wenya Yin

**Affiliations:** ^1^ West China School of Public Health and West China Fourth Hospital, Sichuan University Chengdu China; ^2^ State Key Laboratory of Biotherapy and Cancer Center West China Hospital, Sichuan University Chengdu China; ^3^ Department of Clinical Nutrition Sichuan Provincial People's Hospital, University of Electronic Science and Technology of China Chengdu China; ^4^ Guizhou Jinqianguo Biotechnology Co. Ltd. Bijie China

**Keywords:** gut microbiota, Nrf2/HO‐1/NQO1 signaling pathway, oxidative stress, pulmonary fibrosis, *Rosa roxburghii* fermentation broths

## Abstract

Pulmonary fibrosis (PF) is a chronic and progressive lung disease, and oxidative stress plays a critical role in its pathogenesis. *Rosa roxburghii* Tratt, known for its anti‐inflammatory and antioxidant properties, has been shown to alleviate fibrosis. This study aimed to explore whether two *Rosa roxburghii* fermentation broths (RRFBs) (with different proportions) could attenuate bleomycin (BLM)‐induced PF in mice and to elucidate the molecular mechanisms. The results revealed that RRFBs reduced structural lung damage, collagen deposition, and lung inflammation. RRFBs also suppressed fibrotic markers (Collagen I, Vimentin, and α‐SMA) while enhancing epithelial marker E‐cadherin expression. Additionally, RRFBs alleviated BLM‐induced oxidative stress and apoptosis by activating the Nrf2/HO‐1/NQO1 signaling pathway and facilitating Nrf2 nuclear translocation. Furthermore, RRFBs attenuated the BLM‐induced changes in the gut microbiota; in particular, they decreased the abundance of the pathogenic bacterium *Proteus* and increased the abundance of the probiotics *Ileibacterium* and *Dubosiella*. Spearman correlation analysis revealed a strong association between oxidative stress inhibition and gut microbiota composition. These results indicated that RRFBs could exert lung‐protective effects by inhibiting oxidative stress and alleviating intestinal disturbances.

## Graphical abstract

RRFBs exert a lung‐protective effect by inhibiting oxidative stress and alleviating intestinal disturbances. Our results revealed that RRFBs attenuated BLM‐induced PF in mice. With respect to the underlying mechanisms, RRFBs ameliorated inflammatory responses, EMT, oxidative stress, and apoptosis by activating the Nrf2/HO‐1/NQO1 signaling pathway. They also attenuated alterations in the gut microbiota in PF; in particular, RRFBs decreased the relative abundance of the pathogenic bacterium *Proteus* while increasing the relative abundance of the probiotics *Ileibacterium* and *Dubosiella*.

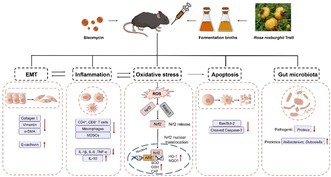

## 1 Introduction

Pulmonary fibrosis (PF) stands as a chronic, severe, and irreversible interstitial disease of the lungs (Lederer and Martinez 2018). As alveolar tissue is gradually replaced by fibrotic scarring, air exchange within the alveoli is compromised, and lung compliance is reduced, resulting in progressive respiratory insufficiency and ultimately demise (Richeldi et al. 2017). Idiopathic pulmonary fibrosis (IPF) has an unknown etiology and is considered to have the most severe manifestations among interstitial lung diseases and the worst clinical outcomes (Selvarajah et al. 2023), with a median survival time of 3–5 years after diagnosis for patients (Podolanczuk et al. 2023). The etiology of IPF includes genetic factors such as mutations in the TERT and TP53 genes, environmental factors such as smoking and occupational exposure, and immune disorders (Saha and Talwar 2024). Despite significant advances in research, the exact etiology and pathogenesis of IPF still need to be further explored. Following recurring outbreaks and the propagation of COVID‐19, a sizable proportion of patients have been diagnosed with PF lesions post‐recovery (Sturgill et al. 2023). A meta‐analysis conducted in 2022 found that 44.9% of COVID‐19 survivors have PF (Hama Amin et al. 2022). Therefore, PF has emerged as a significant global healthcare burden. The United States Food and Drug Administration has only approved pirfenidone and nintedanib for use against PF. However, the efficacies of these drugs are limited, as they only slow disease progression and do not completely abrogate PF; furthermore, they are associated with various side effects (Spagnolo et al. 2021). Consequently, it is necessary to identify safe and effective therapies to halt the advancement of PF.

The pathogenesis of PF is complicated and multifactorial. This process primarily involves the intensification of the epithelial‐mesenchymal transition (EMT) and extracellular matrix (ECM) deposition, induced by alveolar epithelial inflammatory damage. EMT is a pathophysiological process that triggers alveolar epithelial cell apoptosis, the accumulation of fibroblasts/myofibroblasts, and ECM deposition (Moss et al. 2022). More and more data point to oxidative stress as a significant player in the etiopathogenesis of PF (Cheresh et al. 2013). Oxidative stress may trigger PF by damaging epithelial cells and increasing apoptosis (Ornatowski et al. 2020). Nuclear factor erythroid 2‐related factor 2 (Nrf2) plays an essential part in the antioxidant response (Niture et al. 2014). By activating target genes associated with antioxidant response elements (ARE), like heme oxygenase‐1(HO‐1) and NAD(P)H: quinone oxidoreductase 1 (NQO1), Nrf2 inhibits the progression of EMT and alleviates the pathological state of PF (Wang et al. 2022c). As a result, decreasing oxidative stress, diminishing the inflammatory damage, and suppressing the EMT may be useful therapeutic methods for PF.

Gut microbiota balance is critical for sustaining overall health, immunity, tissue homeostasis, and nutritional metabolism. Recent evidence has shown a significant link between gut microbiota and respiratory diseases (Budden et al. 2017). Specific microbial taxa contribute to a healthy immune system by lowering inflammatory reactions and staying away from pulmonary illness. The relationship between the gut and the lung is described as the “gut‐lung axis” (He et al. 2017). The progression of PF may be connected with the dysregulation of the intestinal microbial flora (Gong et al. 2021). Thus, maintaining gut homeostasis is critical for attenuating pulmonary disorders such as PF.

*Rosa roxburghii* Tratt (RRT), a woody plant belonging to the genus *Rosa* within the family *Rosaceae*, exhibits nutritional and medicinal properties (Wang et al. 2021). RRT contains various active substances, including flavonoids, vitamin C, triterpenoids, organic acids, polyphenols, etc. RRT exhibits a range of physiological and pharmacological functions, such as antioxidant, anti‐diabetic, anti‐atherosclerosis, and anti‐inflammatory effects (Ji et al. 2022; Wang et al. 2020). Additionally, RRT can improve the function of the intestinal barrier and diversify the flora (Wang et al. 2023). Studies have confirmed that RRT extract can alleviate renal fibrosis (Zhan et al. 2019), and Rosa sterilis juice can alleviate PF (Wang et al. 2022a). Despite the well‐established nutritional value of RRT, its utilization is limited owing to its spiny exterior, low flesh content, and sour taste (Li et al. 2023b). Currently, RRT is mainly utilized in processed products such as dried fruits, canned fruits, and fruit juice beverages (Li et al. 2022). Fermented plant foods can improve flavor, increase digestibility, and enhance the nutritional value of foods (Leonard et al. 2021). Increasing studies have shown that fermented fruit and vegetable juices have immune‐enhancing, antioxidant, and gastrointestinal protective effects (Hu et al. 2019; Wen et al. 2020). *Rosa roxburghii* fermentation broths (RRFBs) are fermented foods made from RRT. Current research indicates that *Rosa roxburghii* & edible fungus fermentation broth can regulate gut microbiota and improve immunity in immunosuppressed mice (Xu et al. 2023). Fermented RRT fruit juice can improve type 2 diabetes mellitus in mice (Wei et al. 2023). Nevertheless, the effects and mechanisms of RRFBs in PF have not been investigated.

Given the link between PF etiology and oxidative stress, and built on a preliminary study of the active components and antioxidant activity of two proportions of RRFBs, we hypothesized that RRFBs could protect against PF by attenuating oxidative stress. In the current research, we established a mouse model of bleomycin (BLM)‐induced PF and systematically examined the impacts of RRFBs and mechanisms of action, incorporating gut microbiota analysis. This study provides proof of the lung‐protective benefits of RRFBs, as well as novel suggestions for reasonable RRT use.

## 2 Materials and Methods

### 2.1 Composition and Antioxidant Capacity of RRFBs

#### 2.1.1 Component Analysis of the RRFBs

Two proportions of RRFBs were prepared by Sichuan University State Key Laboratory of Biotherapy and Guizhou Jinqianguo Biotechnology Co. Ltd., and were named JIEBEN No. 1 (JB1) and JIEBEN No. 3 (JB3). JB1 and JB3 were tested by Guangzhou Quality Supervision and Testing Institute (ID: 2020‐03‐0132, 2022‐03‐0030). JB1 was obtained by mixing RRT: Honey: *Paeonia lactiflora* Pall. root: *Paeonia lactiflora* Pall. flowe, and *Citrus limon* (L.) Burm. f. at a ratio of 2:2:1:0.3:0.3. JB3 was obtained by mixing RRT: Honey: *Phyllanthus emblica* L.: *Gastrodia elata* Bl, and *Citrus limon* (L.) Burm. f. at a ratio of 2:2:1:0.1:0.3, followed by the addition of *Saccharomyces cerevisiae* , fermentation at room temperature for 360 d, and filtering.

Based on parameters established in our prior experiments (Zhou et al. 2024), 1 mL of RRFBs was diluted with methanol to achieve a total of 10 mL. This solution was filtered via a 0.22 μm membrane after being ultrasonicated for 30 min. HPLC‐Q‐Exactive Orbitrap‐MS (Thermo Fisher, Dreieich, Germany) facilitated the identification of potential bioactive compounds within RRFBs. Visualization was executed via FreeStyle 1.4 and Origin 2022, while compositional analysis and matching were conducted through Compound Discoverer 3.3.2.31, with mzVault and mzCloud databases (Figure S1 and Table S1).

The total flavonoids in RRFBs were measured according to the NaNO_2_‐Al (NO_3_)_3_‐NaOH method (He et al. 2016). The absorbance was examined spectrophotometrically at 510 nm. The standard curve was computed using rutin, and the total flavonoids were reported as mg rutin equivalent per mL RRFBs (mg RE/mL).

The total polyphenols in RRFBs were measured according to the Folin‐Na_2_CO_3_ method (Xia et al. 2022). The absorbance was examined spectrophotometrically at 765 nm. The standard curve was computed using chlorogenic acid, and the total polyphenols were reported as mg chlorogenic acid equivalent per mL RRFBs (mg CAE/mL).

#### 2.1.2 Determination of the Antioxidant Capacity of RRFBs

The antioxidant capacity of RRFBs was measured through scavenging capacity indices for DPPH, ABTS, and OH· radicals, in accordance with the methodology outlined previously (Wang et al. 2022a). Vitamin C was utilized as a reference standard to compare the antioxidant capability of the two RRFBs.

### 2.2 Materials and Reagents

BLM sulfate (#B107423) was obtained from Aladdin (Shanghai, Chinese). Antibodies against Vimentin (#R1308‐6), E‐cadherin(#EM0502), Bcl‐2(#ET1702‐53), Bax(#ET1603‐34) were purchased from HUABIO (Hangzhou, China). Antibodies against Nrf2 (16396‐1‐AP), Cleaved Caspase‐3 (25128‐1‐AP), HO‐1 (10701‐1‐AP), NQO1 (11451‐1‐AP), and Lamin B1 (12987‐1‐AP) were obtained from Proteintech (Wuhan, China). The antibody against GAPDH (#T0004) was obtained from Affinity Biosciences (OH, USA). The antibody against Collagen I(#ab279711) was obtained from Abcam (Cambridge, UK). Goat anti‐rabbit (#ZB‐2305) and anti‐mouse (#ZB‐2301) with horseradish peroxidase (HRP) conjugate were obtained from ZSGB‐BIO (Beijing, China). Anti‐mouse CD4 PE (#100407), anti‐mouse CD8a FITC (#100705), anti‐mouse CD69 APC (#104513), anti‐mouse F4/80 PE (#123109), anti‐mouse CD11b APC (#101212) and anti‐mouse Gr‐1 FITC (#108405) were obtained from Biolegend (CA, USA). Hydroxyproline (HYP), malondialdehyde (MDA), superoxide dismutase (SOD) and glutathione (GSH) activity assay kits were received from Nanjing Jiancheng Bioengineering Institute (Nanjing, China).

### 2.3 Animals and Experimental Design

Male C57BL/6J mice (7–8 wks, 20–22 g) were purchased from Beijing Vital River Laboratory Animal Technology Co. Ltd. (Beijing, China). All animals were maintained in an SPF isolation environment at 25°C ± 1°C, 40%–60% humidity with a 12‐h cycle of light and dark. Animal tests were authorized by the Ethics Committee of West China Fourth Hospital and West China School of Public Health, Sichuan University in China (license number: Gwll2023144) and adhered to the National Institutes of Health's rules (NIH Publication No. 8023). Following a week of acclimation, all mice were split into 6 groups at random, each consisting of 8–12 mice based on their body weight: (1) Control group (Control), (2) BLM model group (Model), (3) 8 mL/kg JIEBEN No. 1 high‐dose group (JB1‐H), (4) 4 mL/kg JIEBEN No. 1 low‐dose group (JB1‐L), (5) 8 mL/kg JIEBEN No. 3 high‐dose group (JB3‐H), and (6) 4 mL/kg JIEBEN No. 3 low‐dose group (JB3‐L). Sodium pentobarbital (55 mg/kg) was administered intraperitoneally to anesthetize all mice, and BLM (2 mg/kg; Aladdin) was injected intratracheally into all but the control group (which received the same amounts of saline). One day after the injection of BLM, mice in four sample groups were gavaged with the corresponding doses. An equal amount of saline was gavaged to the control and model groups. Finally, all groups of mice were euthanized on Day 14.

### 2.4 Flow Cytometry

About 30 mg of fresh lung tissues were excised and digested at 37°C with 4 mL of collagenase I (1 mg/mL) for 90 min. Following digestion, the sample was centrifuged three times, cleaned in PBS, and then the precipitate was resuspended in 1 mL of PBS. Subsequently, 200 μL of the supernatant was mixed with the corresponding antibodies and kept in the dark at room temperature for 30 min (Xia et al. 2022). Measurements were conducted with an Agilent NovoCyte (CA, USA) and the findings were evaluated visually by NovoExpress 1.6.1.

### 2.5 Histopathological Studies

Lung tissues were soaked with 10% formalin, followed by dehydration, clearing, paraffin embedding, sectioning, and rehydration to prepare pathological sections. Sections were stained with Hematoxylin and eosin (H&E) or Masson's trichrome staining (Biossci, Beijing, China), and the degree of PF was assessed using the Ashcroft scale. H&E staining was utilized to observe pulmonary structures, whereas Masson staining was employed to examine collagen deposition. For immunohistochemistry (IHC) (Biossci, Beijing, China), lung tissues were embedded in paraffin and stained with antibodies against Collagen I, α‐smooth muscle actin (α‐SMA), and E‐cadherin. Lung tissue pathological changes were observed with a 3DHISTECH digital microscope and CaseViewer. The degree of PF and IHC scores were analyzed based on previously reported methodologies.

### 2.6 Western Blot Analysis

Total proteins were isolated from lung tissue utilizing RIPA lysis buffer with 0.1% PMSF. Nuclear and cytoplasmic proteins were isolated using precise protocols provided by Beyotime (Shanghai, China). Protein concentrations were examined through the bicinchoninic acid method, followed by denaturation with SDS and heating to 100°C for 8 min. Then the separation of proteins via SDS‐polyacrylamide gel electrophoresis was carried out and transferred to membranes made of polyvinylidene difluoride. The membranes were immersed in 5% skim milk for 1 h, then incubated with primary antibodies overnight at 4°C. A day later, the membranes were incubated at room temperature for 1 h with HRP‐conjugated secondary antibodies. Visualization was executed with an enhanced chemiluminescence kit. Gray scale values of protein bands were identified using ImageJ, and the target band's ratio to GAPDH was utilized for semi‐quantitative analysis.

### 2.7 Quantitative Polymerase Chain Reaction (qPCR) Analysis

Total RNA was collected from mice lung tissues with an isolation kit (Foregene, Chengdu, China). The concentration of total RNA was confirmed with a NanoDrop 2000 nm spectrophotometer (Thermo, MA, USA). Subsequently, RNA samples were reverse‐transcribed into cDNA through the ABScript II cDNA First‐Strand Synthesis Kit (ABclonal, Wuhan, China). Relative gene expression was determined with the Taq Pro Universal SYBR qPCR Master Mix (Vazyme, Nanjing, China). Data analysis was conducted employing the 2^−∆∆CT^ method, with normalization to GAPDH. Sangon Biotech (Shanghai, China) manufactured the primers, and their sequences are presented in Table S2.

### 2.8 Immunofluorescence Assay

Sections were deparaffinized in xylene, rehydrated in ethanol, and boiled in citrate buffer (0.01 M, pH 6.0). Sections were covered with 10% serum for 30 min at 37°C and treated with an antibody against Nrf2 for an overnight period at 4°C. A day later, sections were treated with goat anti‐rabbit IgG H&L secondary antibody for 45 min at 37°C. Nrf2 nuclear translocation was detected using a FITC filter and a Carl Zeiss fluorescent microscope at a magnification of 200× and analyzed by ImageJ.

### 2.9 Measurement of HYP, MDA, GSH, and SOD Levels

Lung tissues were processed into 10% homogenates, and HYP, MDA, GSH, and SOD levels were assayed utilizing specifically designed commercially available kits, following the manufacturer's guidelines.

### 2.10 Gut Microbiota Analysis

Fecal samples were obtained from mice in the Control, Model, JB1‐H, and JB3‐H groups. Genomic DNA extraction was conducted with a DNA extraction toolkit (Qiagen, Hilden, Germany). Based on preliminary quantification outcomes from electrophoresis, PCR products were next quantified with the QuantiFluor‐ST Blue Fluorescence Quantification System (Promega, Beijing, China). Subsequently, the TruSeq DNA Sample Prep Kit (Illumina, CA, USA) was used to create sequencing libraries, followed by high‐throughput sequencing. Quality checking was carried out with QIIME v1.9.1. Clustering of operational taxonomic units (OTUs) was performed, and annotation was done with the UCLUST taxonomy and the SILVA138 database.

### 2.11 Statistical Analysis

The results were analyzed utilizing GraphPad Prism 9 and ImageJ, and presented as means ± standard deviation (SD). Group differences were assessed using an independent sample *t*‐test or one‐way analysis of variance (ANOVA), followed by Dunnett post hoc tests. Values of *p* < 0.05 indicated significant differences. *p < 0.05, **p < 0.01, ***p < 0.001 vs. the control group. ^#^ *p* < 0.05, ^##^ *p* < 0.01, ^###^ *p* < 0.001 vs. the model group.

## 3 Results

### 3.1 Components and Antioxidant Capacity Analysis of RRFBs

We employed HPLC‐Q‐Exactive Orbitrap‐MS for the analysis of components within two RRFBs, resulting in the identification of 91 substances with a mass spectral match exceeding 90%, including organic acids, flavonoids, amino acids, and polyphenols. The results of the total ion chromatographic determination can be found in Figure S1 and Table S1. Flavonoids and polyphenols constitute functional components in fruits, exhibiting a wide range of biological activities. We used a colorimetric method to evaluate flavonoids and polyphenols in the two RRFBs. As shown in Table 1, the total flavonoid content was 6.53 ± 0.14 mg RE/mL and the total polyphenol content was 14.49 ± 0.64 mg CAE/mL in JB1, while the total flavonoid content in JB3 was 3.51 ± 0.10 mg RE/mL and the total polyphenol content was 49.36 ± 0.89 mg CAE/mL. Free radical scavenging capacity is one of the core mechanisms of antioxidant action; thus, we used DPPH, ABTS, and OH radical‐scavenging assays to assess the antioxidant capacity of the two RRFBs, as shown in Table 2. The IC_50_ values of JB1 for DPPH, ABTS, and OH radical‐scavenging activity were 11.46 ± 0.18 μL/mL, 3.67 ± 0.06 μL/mL, and 21.37 ± 0.80 μL/mL, respectively. The IC_50_ values of JB3 in DPPH, ABTS, and OH radical‐scavenging activity were 3.32 ± 0.03 μL/mL, 1.01 ± 0.04 μL/mL, and 24.17 ± 1.20 μL/mL, respectively. The antioxidant capacity of both RRFBs was much stronger than that of Vitamin C. This demonstrates that both RRFBs possess considerable antioxidant capacity. By *t*‐test, the contents of total flavonoids and total polyphenols of the two samples were significantly different. The total polyphenol content was approximately 3.41 times higher in JB3 than in JB1, and the total flavonoid content of JB1 is also 1.86 times that of JB3. The total polyphenol content of JB3 is more significantly higher, and polyphenol compounds generally dominate the active ingredient because they have a wider range of biological activities (Rudrapal et al. 2024). Taken together, JB3 exhibits higher active ingredient concentrations and antioxidant activity than those of JB1.

**TABLE 1:** Total flavonoids and total polyphenols content in RRFBs.

	Total polyphenols (mg RE/mL)	Total flavonoids (mg CAE/mL)
JB1	14.49 ± 0.64^a^	6.53 ± 0.14^a^
JB3	49.36 ± 0.89^b^	3.51 ± 0.10^b^

*Note:* Different letters in the same column (a, b) represent significant differences (*p* < 0.05).

**TABLE 2:** Analysis of antioxidant capacity in RRFBs.

			IC_50_
	DPPH^+^	ABTS^+^	·OH
JB1 (μL/mL)	11.46 ± 0.18^b^	3.67 ± 0.06^b^	21.37 ± 0.80^b^
JB3 (μL/mL)	3.32 ± 0.03^c^	1.01 ± 0.04^c^	24.17 ± 1.20^b^
Vitamin C (μL/mL)	107.57 ± 1.18^a^	54.77 ± 0.74^a^	149.20 ± 4.37^a^

*Note:* Different letters in the same column (a–c) represent significant differences (*p* < 0.05).

### 3.2 RRFBs Ameliorated BLM‐Induced PF in Mice

**FIGURE 1:**
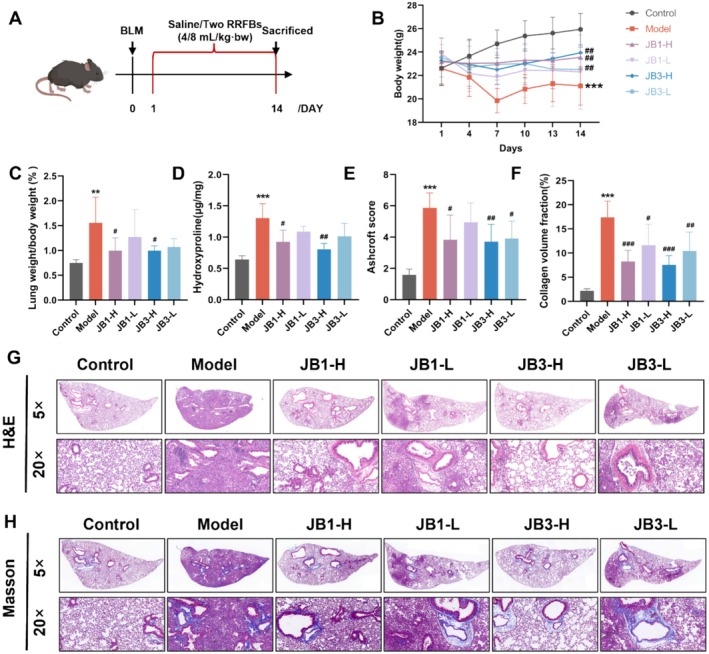
RRFBs ameliorated BLM‐induced PF in mice. (A) Animal experimental design. (B) Body weight of mice. (C) Lung weight coefficient of mice. (D) The hydroxyproline content in the mice lung tissues. (E) Ashcroft score of lung tissues. (F) Collagen volume fraction. (G) HE staining of lung tissues, magnification: 5×and 20×. (H) Masson's trichrome staining of lung tissues, magnification: 5×and 20×. Data are presented as mean ± SD. *p < 0.05, **p < 0.01, ***p < 0.001 compared to the control group. ^#^ *p* < 0.05, ^##^ *p* < 0.01, ^###^ *p* < 0.001 compared to the model group.

By injecting BLM into the trachea, a mouse model of PF was created in order to investigate the functions of two RRFBs in PF. The overall body weight of control mice showed an increasing trend, whereas the model group showed a significant decrease. This decrease was attenuated in the RRFBs intervention groups, and the final body weight of the JB3‐H group was more similar to that of the control group (Figure 1B). Additionally, the model group's lung‐to‐weight coefficient and HYP content were notably higher than those of the control group (Figure 1C,D). Following intervention with RRFBs, the lung weight coefficient, HYP content, Ashcroft score, and area of collagen deposition decreased (Figure 1E,F). H&E staining showed that the control group had intact tissue structures, with no inflammatory cell infiltration or alveolar septal thickening. However, the model group showed disorganized alveolar structures, thickened alveolar septa, and significantly increased inflammatory cell infiltration supporting the successful establishment of the PF model (Figure 1G). Masson staining results showed substantial blue fibrous tissue and more extensive collagen deposition in the model group (Figure 1H). The high‐dose intervention groups (JB1‐H and JB3‐H) were superior to the low‐dose groups (JB1‐L and JB3‐L) in alleviating BLM‐induced pulmonary injury and fibrosis.

### 3.3 RRFBs Reduced Lung Inflammation After BLM‐Induced

**FIGURE 2:**
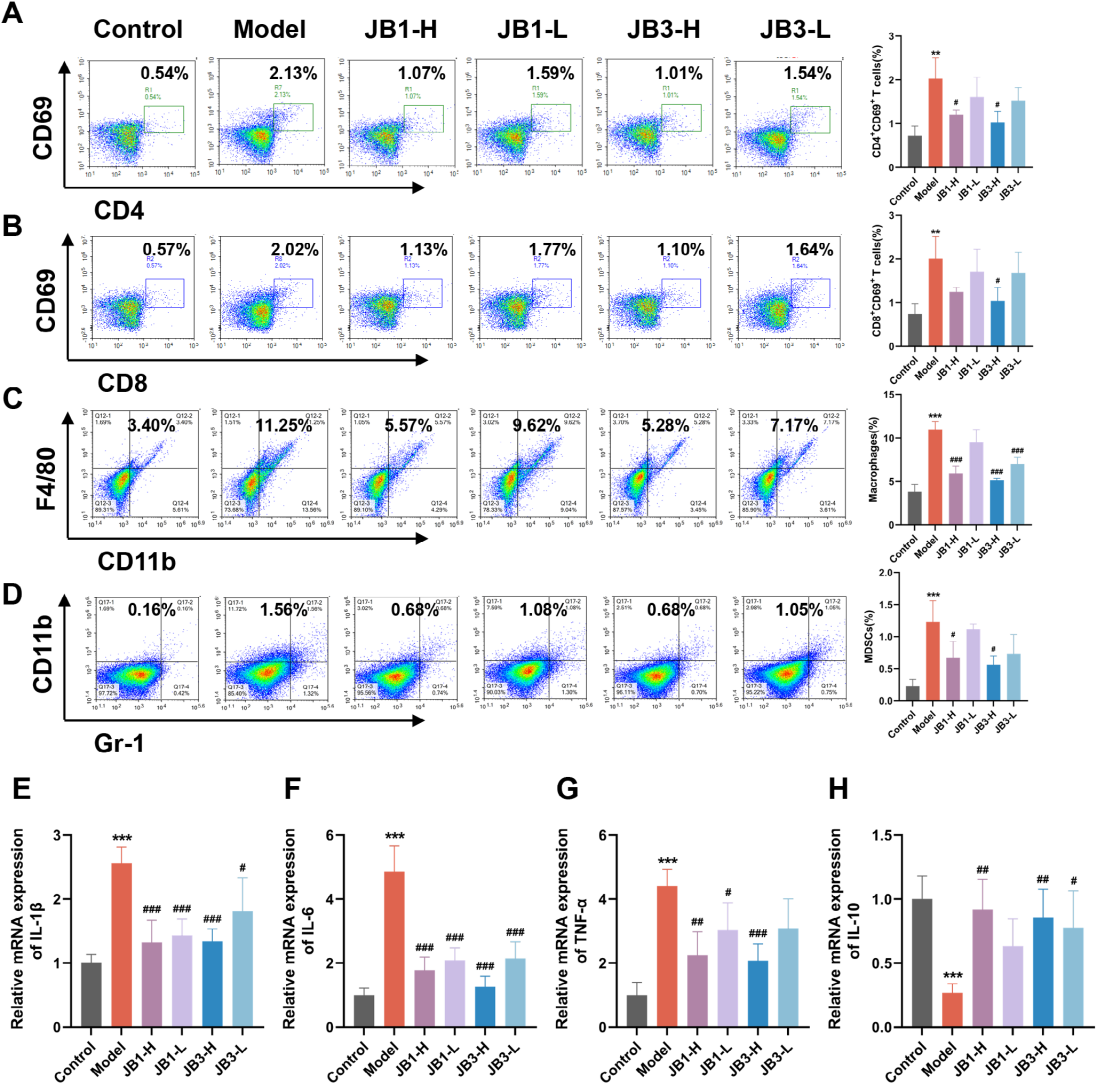
RRFBs reduced the lung inflammation after BLM‐induced. (A) The expression of CD4 ^+^ CD69^+^ T cells. (B) The expression of CD8 ^+^ CD69^+^ T cells. (C) The expression of macrophages. (D) The expression of MDSCs. (E–H) Relative mRNA expression of IL‐1β, IL‐6, TNF‐α, and IL‐10. Data are presented as mean ± SD. Data are presented as mean ± SD. *p < 0.05, **p < 0.01, ***p < 0.001 compared to the control group. ^#^ *p* < 0.05, ^##^ *p* < 0.01, ^###^ *p* < 0.001 compared to the model group.

To assess the effect of RRFBs on lung inflammation during PF, we examined inflammatory cells in mice lung tissues using flow cytometry. As shown in Figure 2A–D there were significantly more CD4^+^ CD69^+^ T cells, CD8^+^ CD69^+^ T cells, macrophages, and myeloid‐derived suppressor cells (MDSCs) in the model group compared to the control group, and these increases were attenuated by RRFBs. The effects of RRFBs were more pronounced in the high‐dose groups (JB1‐H and JB3‐H) than in the low‐dose groups (JB1‐L and JB3‐L), with JB3‐H causing the most significant reduction in inflammation. Furthermore, we used qPCR to measure inflammatory indicators in lung tissues (Figure 2E–G), mRNA expression levels of pro‐inflammatory cytokines interleukin‐1β (IL‐1β), interleukin‐6 (IL‐6), and tumor necrosis factor‐α (TNF‐α) were notably higher in the model group compared with the control group and were reduced to various extents in the RRFBs intervention groups. Conversely, levels of the anti‐inflammatory cytokine interleukin‐10 (IL‐10) were notably lower in the model group, and these differences were attenuated in the RRFBs intervention groups. The JB1‐H and JB3‐H groups were more effective at lowering pro‐inflammatory cytokine levels and boosting anti‐inflammatory cytokine levels. These results indicated that RRFBs could regulate immune cell infiltration and reduce lung inflammation after BLM‐induced.

### 3.4 RRFBs Inhibited BLM‐Induced EMT in Mice

**FIGURE 3:**
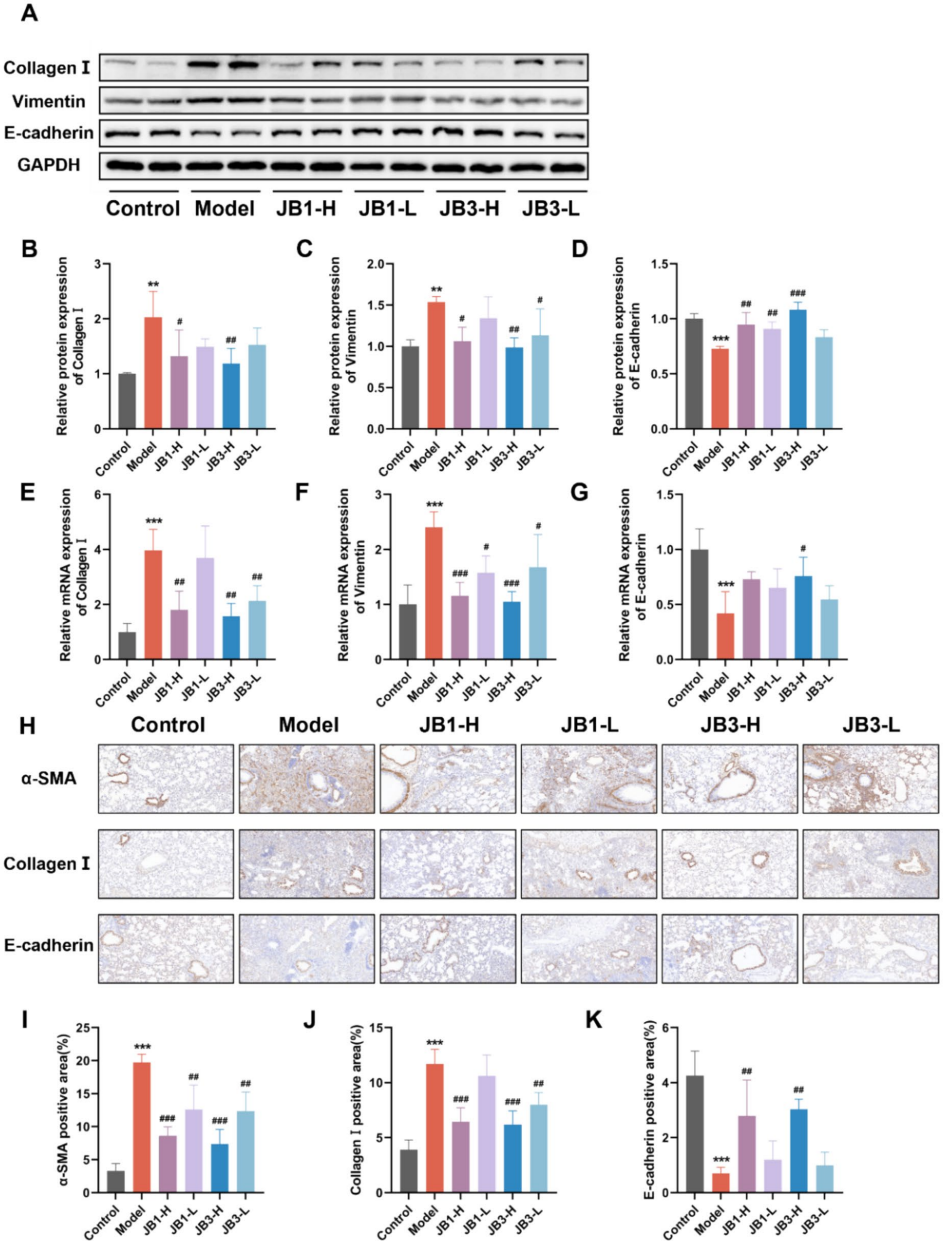
RRFBs inhibited BLM‐induced EMT in mice. (A) Western blot analysis of Collagen I, Vimentin, and E‐cadherin. (B‐D) Relative protein expression of Collagen I, Vimentin, and E‐cadherin. (E‐G) Relative mRNA expression of Collagen I, Vimentin, and E‐cadherin. (H) Immunohistochemical staining of α‐SMA, Collagen I, and E‐cadherin in lung tissues, magnification: 20×. (I, J) Positive area of α‐SMA, Collagen I, and E‐cadherin. Data are presented as mean ± SD. *p < 0.05, **p < 0.01, ***p < 0.001 compared to the control group. ^#^ *p* < 0.05, ^##^ *p* < 0.01, ^###^ *p* < 0.001 compared to the model group.

EMT is a key mechanism underlying fibrosis and the development of PF. We verified the impact of RRFBs on EMT‐related markers in BLM‐induced PF. Western blot results (Figure 3A–D) revealed that the expression of Collagen I and Vimentin was much higher in the model group compared with the control group, whereas E‐cadherin expression was significantly lower. High dosages of RRFBs (JB1‐H and JB3‐H) dramatically reduced Collagen I and Vimentin expression while increasing E‐cadherin expression; the JB3‐H group had the best effect. Additionally, qPCR revealed that the relative mRNA expression levels of Collagen I and Vimentin were elevated, whereas the expression level of E‐cadherin was significantly lowered after BLM treatment, and, on the contrary, RRFBs treatment attenuated the tendency of BLM‐induced mRNA expression of EMT markers, and the effects were most significant in the JB3‐H group (Figure 3E–G). IHC staining of lung tissues yielded similar results (Figure 3H–K), demonstrating that RRFBs decreased α‐SMA and Collagen I expression and increased E‐cadherin expression. These findings suggested that RRFBs, especially JB3‐H, exert anti‐PF effects by inhibiting BLM‐induced EMT in mice.

### 3.5 RRFBs Reduced BLM‐Induced Apoptosis of Alveolar Epithelial Cells

**FIGURE 4:**
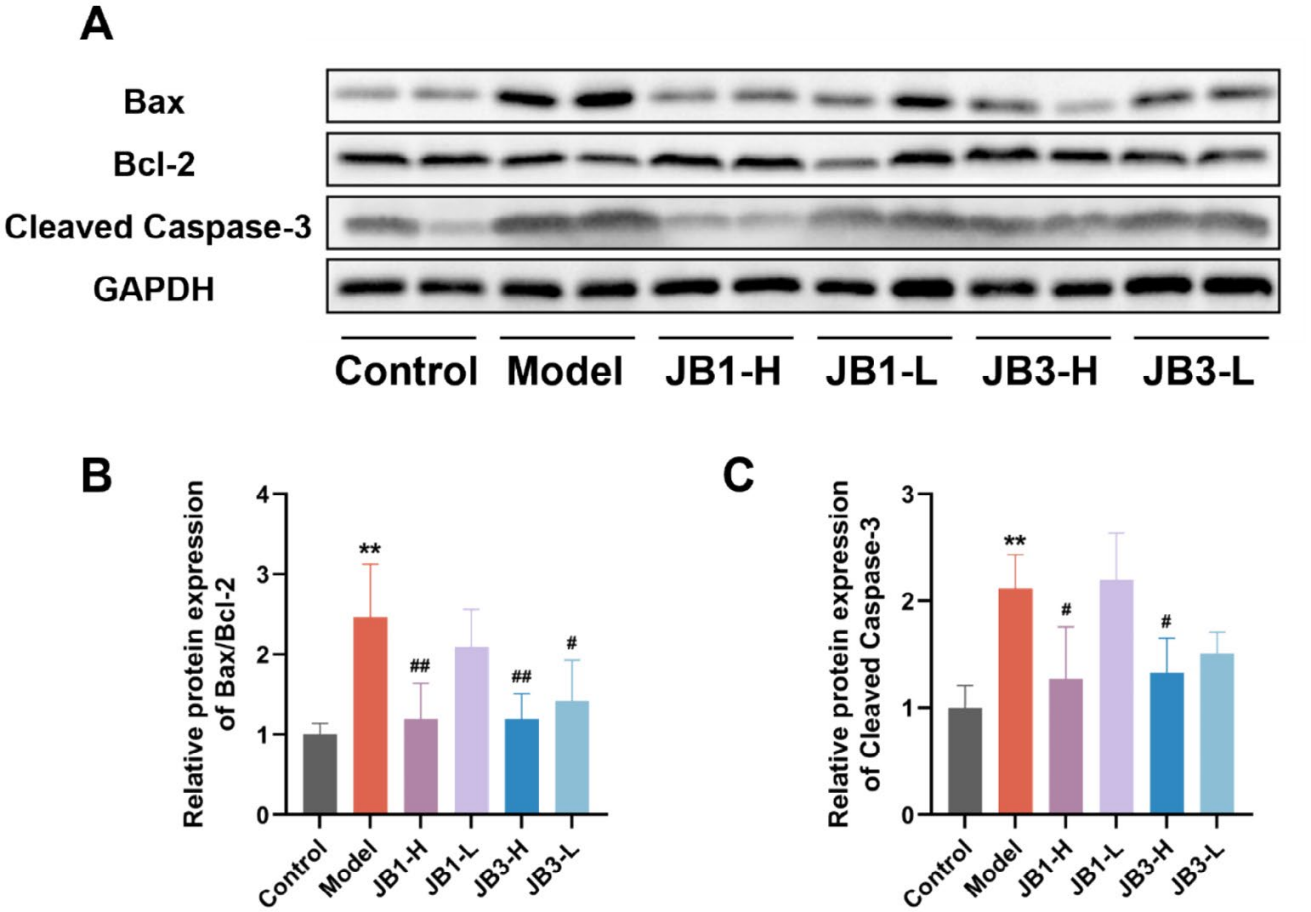
RRFBs reduced BLM‐induced apoptosis of alveolar epithelial cells. (A) Western blot analysis of Bax, Bcl‐2, and Cleaved Caspase‐3. (B, C) Relative protein expression of Bax/Bcl‐2 and Cleaved Caspase‐3. Data are presented as mean ± SD. *p < 0.05, **p < 0.01, ***p < 0.001 compared to the control group. ^#^ *p* < 0.05, ^##^ *p* < 0.01, ^###^ *p* < 0.001 compared to the model group.

EMT triggers apoptosis in alveolar epithelial cells. Accordingly, the expression of apoptosis‐related proteins in each group was further examined using western blot (Figure 4A–C). In the model group, apoptotic protein Bax expression was dramatically raised, anti‐apoptotic protein Bcl‐2 expression was dramatically reduced, and Cleaved Caspase‐3 expression was notably enhanced compared with the control group. RRFBs intervention downregulated Bax/Bcl‐2 and Cleaved Caspase‐3, hence preventing the BLM‐induced increase in alveolar epithelial cells' apoptosis, and the intervention effect in the high‐dose groups (JB1‐H and JB3‐H) was stronger than that in the low‐dose groups (JB1‐L and JB3‐L). These results indicated that RRFBs could prevent PF by inhibiting apoptosis.

### 3.6 RRFBs Inhibited the Expression of Oxidative Stress‐Related Markers in BLM‐Induced Mice

**FIGURE 5:**
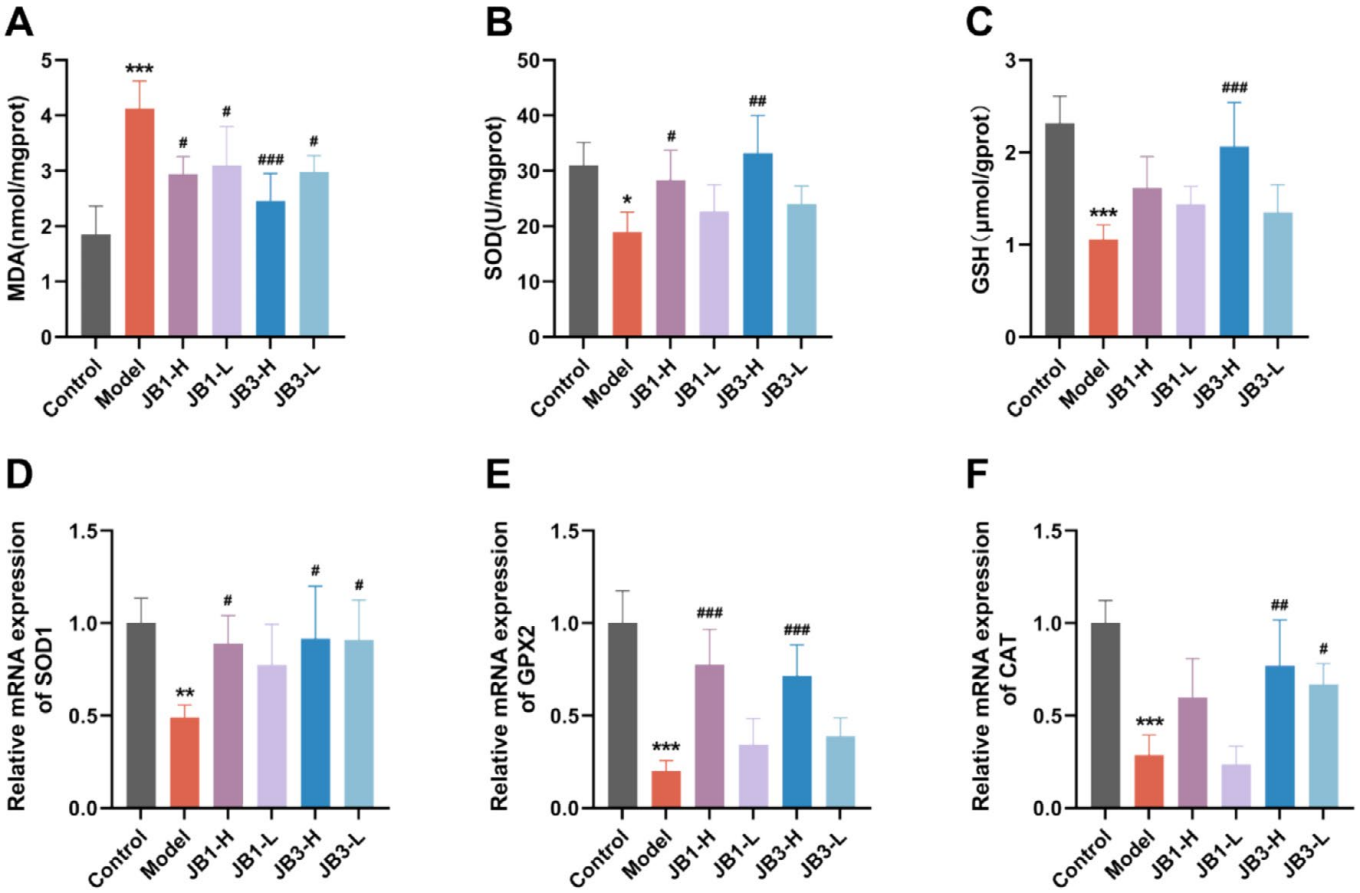
RRFBs inhibited BLM‐induced oxidative stress in mice. (A) MDA level. (B) SOD activity. (B) GSH level. (D–F) Relative mRNA expression of SOD1, GPX2, and CAT. Data are presented as mean ± SD. *p < 0.05, **p < 0.01, ***p < 0.001 compared to the control group. ^#^ *p* < 0.05, ^##^ *p* < 0.01, ^###^ *p* < 0.001 compared to the model group.

The levels of MDA, SOD, and GSH in mice lung tissues were assessed to determine whether the effects of RRFBs on BLM‐induced PF were related to antioxidant capacity. As shown in Figure 5, MDA levels (Figure 5A) were significantly higher, and SOD (Figure 5B) and GSH (Figure 5C) levels were dramatically lower in the model group compared with the control group. RRFBs elevated SOD and GSH levels and diminished MDA overproduction. Particularly, the JB3‐H group showed the greatest reduction in BLM‐induced oxidative stress. Furthermore, qPCR analysis of the mRNA expression of SOD1, GPX2, and CAT yielded consistent results (Figure 5D–F). In contrast with the control group, mRNA expression of SOD1, GPX2, and CAT was notably down‐regulated in the model group, and these decreases were attenuated to varying degrees in the RRFBs sample groups, with significant upregulation in the JB3‐H group (consistent with the stronger antioxidant effect of JB3 in the antioxidant capacity analysis in vitro). These findings suggested that RRFBs could inhibit BLM‐induced oxidative stress through antioxidant activity.

### 3.7 RRFBs Activated the Nrf2/HO‐1/NQO1 Signaling Pathway

**FIGURE 6:**
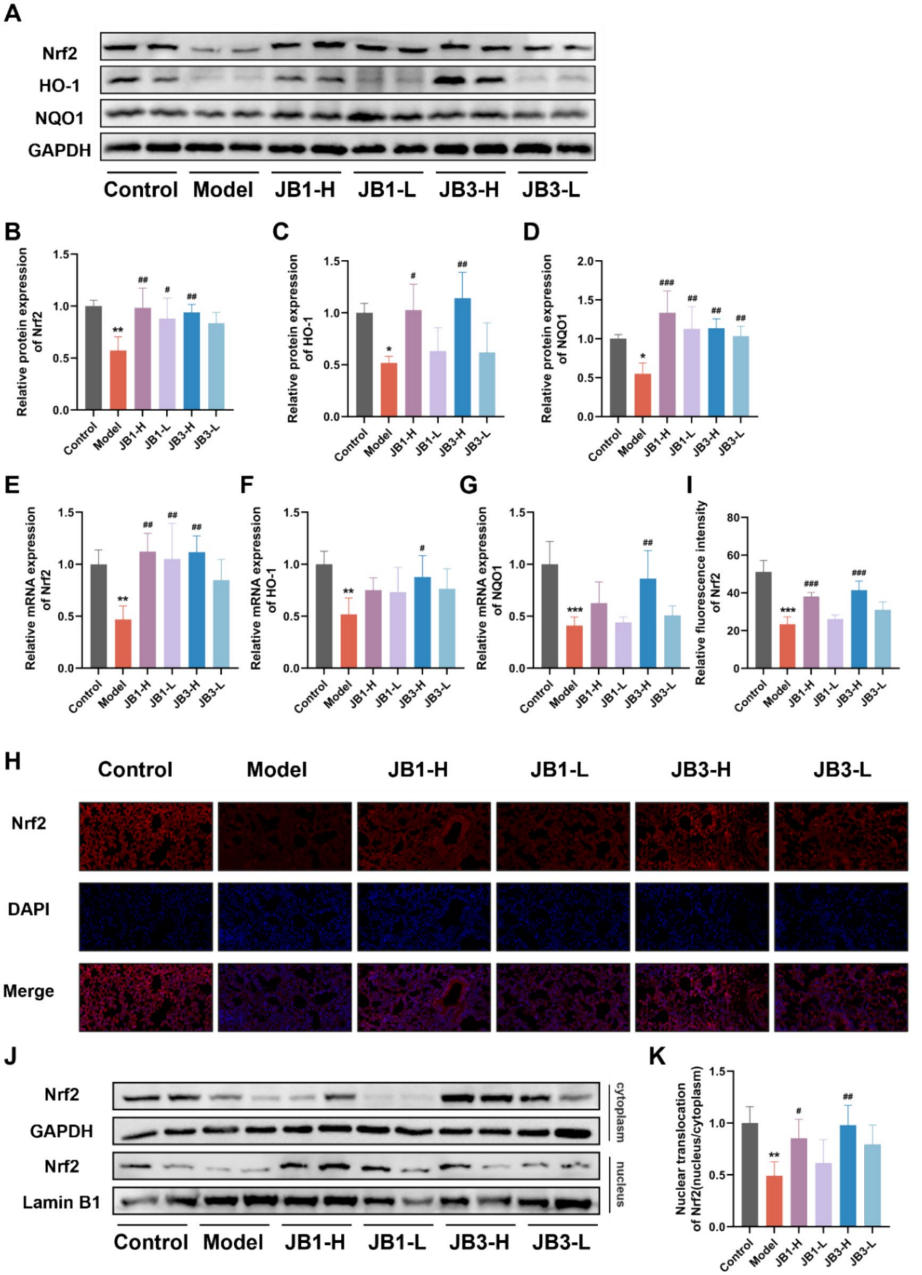
RRFBs activated the Nrf2/HO‐1/NQO1 signaling pathway. (A) Western blot analysis of Nrf2, HO‐1 and NQO1. (B–D) Relative protein expression of Nrf2, HO‐1, and NQO1. (E‐G) Relative mRNA expression of Nrf2, HO‐1, and NQO1. (H) Immunofluorescence staining of Nrf2, magnification:40×. (I) Relative fluorescence intensity of Nrf2. (J, K) Nuclear translocation of Nrf2 by western blot analysis. Data are presented as mean ± SD. *p < 0.05, **p < 0.01, ***p < 0.001 compared to the control group. ^#^ *p* < 0.05, ^##^ *p* < 0.01, ^###^ *p* < 0.001 compared to the model group.

To better understand the molecular processes by which RRFBs attenuate BLM‐induced oxidative stress, we investigated the protein and gene expression of Nrf2 and its downstream genes (HO‐1/NQO1). Western blot analysis revealed that the model group exhibited significantly lower protein expression levels of Nrf2, HO‐1, and NQO1 than the control group (Figure 6A–D), whereas RRFBs intervention exhibited an opposite effect. qPCR analysis showed that the mRNA expression of Nrf2, HO‐1, and NQO1 was down‐regulated in the model group compared with the control group, and Nrf2, HO‐1, and NQO1 expression was differentially raised in the RRFBs groups (Figure 6E–G). Immunofluorescence was used to further examine the changes in Nrf2 in mice lung tissues (Figure 6H,I), revealing that the positive fluorescence signal and nuclear localization of Nrf2 in the model group were observably fewer than those in the control group, while RRFBs notably increased the nuclear localization of Nrf2. RRFBs promoted Nrf2 nuclear translocation (Figure 6J,K). These results suggested that RRFBs might ameliorate BLM‐induced oxidative stress by up‐regulating Nrf2 expression and activating downstream genes (HO‐1/NQO1), contributing to the mitigation of PF.

### 3.8 RRFBs Alleviated the Disturbance of Gut Microbiota in BLM‐Induced Mice

**FIGURE 7:**
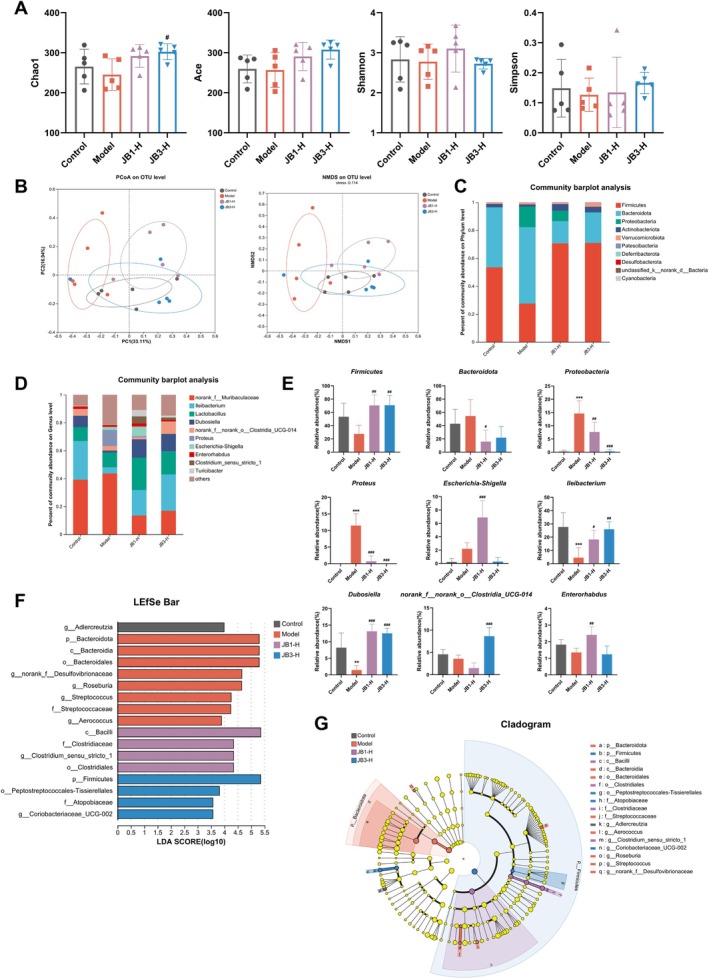
RRFBs alleviated the disturbance of gut microbiota in BLM‐induced mice. (A) Chao1, Ace, Shannon, and Simpson indices. (B) PCoA and NMDS analysis. (C) Microbiota compositions at the phylum level. (D) Microbiota compositions at the genus level. (E) Analysis of bacterial flora with a significant difference at the phylum and genus levels. (F) Histogram of LDA value distribution. (G) Cladogram of LEfSe analysis. Data are presented as mean ± SD. *p < 0.05, **p < 0.01, ***p < 0.001 compared to the control group. ^#^ *p* < 0.05, ^##^ *p* < 0.01, ^###^ *p* < 0.001 compared to the model group.

To explore whether high doses of RRFBs could alleviate gut microbiota disorders in BLM‐induced mice, we performed 16S rDNA sequencing analysis. Indices of α‐diversity, Chao1 and Ace, were utilized to assess community richness (Figure 7A). The model group reduced the Chao1 and Ace indices relative to the control group. These BLM‐induced decreases in richness were attenuated by RRFBs, and the Chao1 index in the JB3‐H group was notably higher relative to the model group. In the community diversity study, the Shannon and Simpson indices were reduced in the model group; however, the differences were not of statistical significance. Additionally, β‐diversity was evaluated using a principal coordinates analysis (PCoA) and nonmetric multidimensional scaling (NMDS) (Figure 7B). The model group was distinguished from the control group, while the RRFBs groups differed significantly from the model group. The microbial composition and structure were similar between the JB3‐H and the control groups. Typically, NMDS is deemed to have interpretative significance when stress is < 0.2. The NMDS stress value of 0.114 observed in this experiment supports the validity of the results. These results suggested that RRFBs increased the richness and diversity of gut microbiota in BLM‐induced mice.

We then analyzed the compositional structure of the gut microbiota between the four groups at the phylum and genus levels (Figure 7C–E). At the phylum level, the abundance of *Firmicutes* decreased, and the abundance of *Bacteroidota* and *Proteobacteria* increased in the model group compared to the control group. These trends reversed following RRFBs treatment. At the genus level, the relative abundance of pathogenic bacteria such as *Proteus* and *Escherichia‐Shigella* increased in the model group compared to the control group, while *Ileibacterium*, *Dubosiella*, *norank_f_norank_o_Clostridia_UCG‐014*, and *Enterorhabdus* were less abundant. RRFBs notably reduced the relative abundance of the pathogenic bacterium *Proteus* and enhanced those of probiotics *Ileibacterium* and *Dubosiella*.

Eventually, we conducted a linear discriminant analysis effect size (LEfSe) to determine the dominant microbiota in each group (LDA values > 3, *p* < 0.05) (Figure 7F,G). Linear discriminant analysis (LDA) showed that the dominant species in the control group were *Adlercreutzia*, whereas in the model group, the dominant species were *Bacteroidota*, *Bacteroidia*, *Bacteroidales*, *Desulfovibrionaceae*, *Roseburia*, *Streptococcaceae*, *Streptococcus*, and *Aerococcus*. After treatment with RRFBs, the dominant taxa in the JB1‐H group were *Bacilli*, *Clostridiales*, *Clostridiaceae*, and *Clostridium_sensu_stricto_1*, and the dominant taxa in the JB3‐H group were *Firmicutes*, *Peptostreptococcales‐Tissierellales*, *Atopobiaceae*, and *Coriobacteriaceae_UCG‐002*. These results suggested that RRFBs could regulate BLM‐induced disruptions in the gut microbiota and boost the number of beneficial bacteria, thereby alleviating PF.

### 3.9 Spearman Correlation Analysis Between Oxidative Stress and Gut Microbiota

**FIGURE 8:**
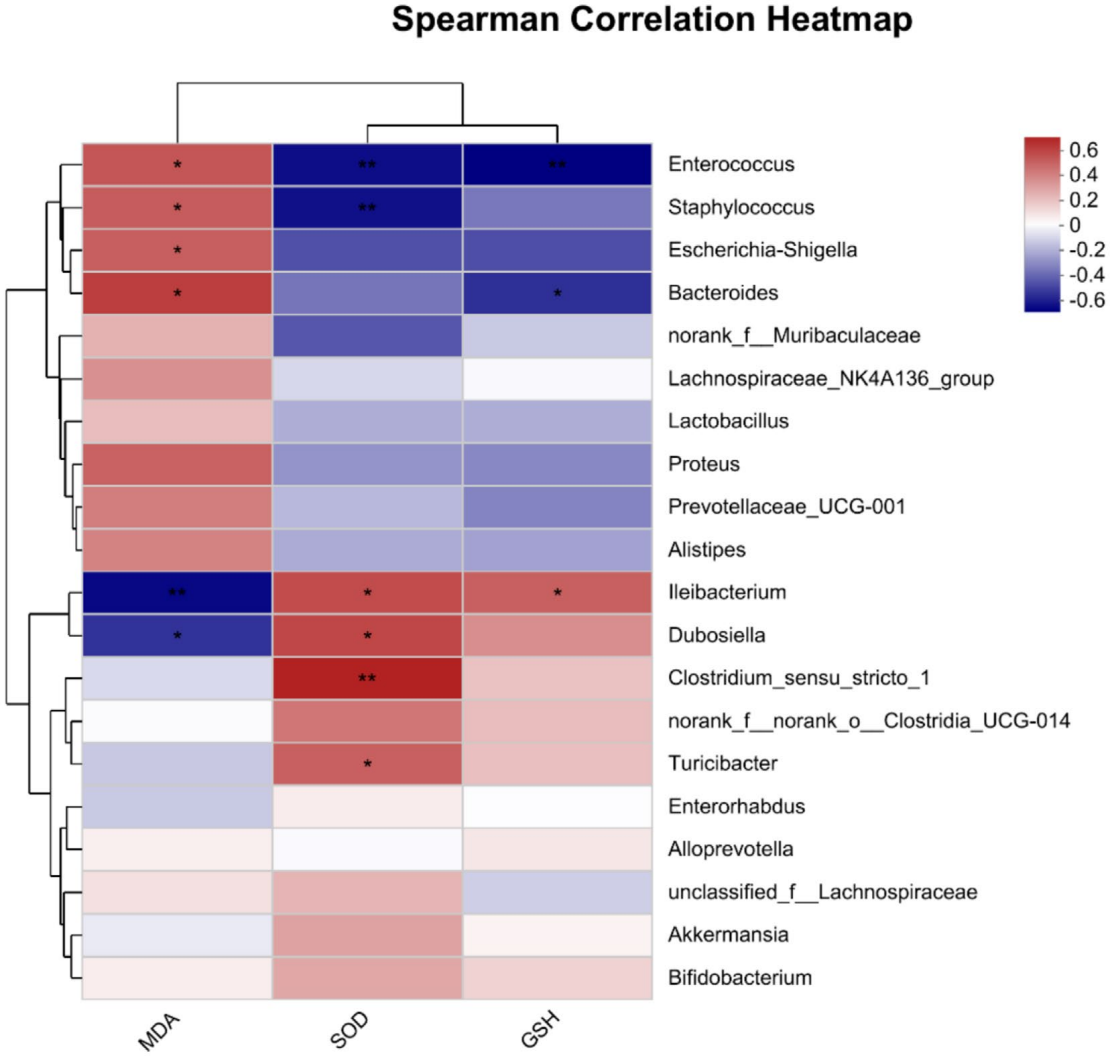
Spearman correlation analysis between oxidative stress and gut microbiota. *p < 0.05; **p < 0.01; ***p < 0.001.

To further explore functional correlations between gut microbiota and oxidative stress‐related indicators in PF, we assessed species in the top 20 with respect to total abundance at the genus level (Figure 8). Spearman correlation analyses revealed that MDA was markedly negatively correlated with probiotic bacteria *Ileibacterium* and *Dubosiella* and markedly positively correlated with pathogenic bacteria *Enterococcus*, *Staphylococcus*, *Escherichia –Shigella*, and *Bacteroides*. SOD had substantial negative correlations with *Enterococcus* and *Staphylococcus*, but significant positive correlations with *Ileibacterium*, *Dubosiella*, *Turicibacter*, and *Clostridium_sensu_stricto_1*. GSH exhibited substantial positive correlations with *Enterococcus* and *Bacteroides* and a significant negative correlation with *Ileibacterium*. Overall, SOD and GSH were positively correlated with probiotic bacteria, but MDA demonstrated a positive correlation with pathogenic bacteria. These findings indicated that the gut microbiota and oxidative stress were closely related.

## 4 Discussion

The hallmark of PF is the continuous and progressive degeneration of lung structures brought on by the deposition of scar tissue (Savin et al. 2022). Given the multifactorial nature of the pathogenesis of PF, drugs with multitargeting capabilities are promising therapeutic strategies. Natural active components found in abundance in RRT include organic acids, flavonoids, and polyphenols. It can be consumed fresh or processed into fruit juices, dried products, canned products, etc. Fermented plant foods are receiving increasing attention owing to their unique flavor and beneficial health effects. Fermentation effectively improves the overall quality of RRT (Li et al. 2023a). The current study found that the total polyphenols and flavonoids content and antioxidant capacity of two formulations of RRFBs far exceeded those of RRT fruits (Wang et al. 2023). The DPPH and ABTS radical‐scavenging activity of JB3 was about 3.50 times higher than that of JB1, while the OH radical scavenging capacity was similar. Additionally, the total polyphenols content of JB3 was about 3.41 times higher than that of JB1. Therefore, JB3 was generally superior to JB1 in terms of its active ingredients and antioxidant capacity. The abundant active ingredients and excellent antioxidant capacity in RRFBs might reduce oxidative stress and thus help to alleviate PF.

BLM is an inducible cytotoxic antitumor drug and is widely used as an inducer in animal models of PF (Della Latta et al. 2015). After BLM modeling, in addition to obvious histopathological damage, the lung‐to‐weight coefficient and HYP were notably elevated in the model group. Intervention with RRFBs attenuated the abnormal accumulation of fibrous tissues and collagen, with superior effects in the high‐dose groups compared to the low‐dose groups. Inflammatory infiltration is a significant pathological feature of PF. Macrophages, MDSCs, and T cells are activated during the early inflammatory injury stage of PF. Studies have demonstrated that T cell levels (e.g., CD4^+^ T and CD8^+^ T cells) in lung tissue are significantly correlated with fibrosis severity. Macrophages are innate immune cells that mediate epithelial damage repair and secrete key pro‐fibrotic factors such as IL‐6 and TNF‐α to exacerbate PF. MDSCs play a role in immunity via suppressive effects on T cells (Desai et al. 2018). The present research discovered that high‐dose RRFBs efficiently reduced CD4^+^ CD69^+^ T cells, CD8^+^ CD69^+^ T cells, macrophages, and MDSCs, with the greatest effects observed in the JB3‐H group. During PF, lung epithelial injury triggers the release of pro‐inflammatory cytokines, leading to increased macrophage and lymphocyte aggregation and fibroblast activation (Xin et al. 2019). In this study, the BLM‐induced group had higher levels of pro‐inflammatory cytokines (IL‐1β, IL‐6, and TNF‐α) and lower levels of the anti‐inflammatory cytokine (IL‐10) compared with the control group, and these BLM‐induced changes were attenuated by RRFBs.

Although the pathogenesis of PF is complex and not completely understood, the EMT process contributes to disease progression. The EMT process involves the conversion of epithelial cells into myofibroblasts, marked by a diminution in epithelial markers like E‐cadherin and increases in mesenchymal markers like collagen, α‐SMA, and vimentin (Nieto et al. 2016). Reduced E‐cadherin expression leads to the loss of epithelial properties and disruption of cellular structural stability, resulting in a transformation to spindle‐shaped mesenchymal cells. The study found that BLM effectively caused EMT, as evidenced by increases in Collagen I, Vimentin, and α‐SMA expression and a diminution in E‐cadherin expression in the model group relative to the control group. Both RRFBs lowered Collagen I, Vimentin, and α‐SMA expression and boosted E‐cadherin expression. Overall, the high‐dose groups were more effective than the low‐dose groups in alleviating EMT, and the greatest inhibitory effect was observed in the JB3‐H group, potentially due to its higher contents of natural products. These results further indicated that RRFBs might inhibit PF by alleviating EMT.

Apoptosis, a process of programmed cell death, is necessary to preserve healthy lung homeostasis and plays a role in the etiology of numerous lung disorders, including PF (Sul et al. 2022). Apoptosis was viewed in the lungs of COVID‐19 patients with severe PF in clinical investigations as well as in TGF‐β1‐induced epithelial cells and an animal model of PF (Wheaton et al. 2016; Xiao et al. 2023). The B‐cell lymphoma/leukemia 2 (Bcl‐2) family is an essential regulator of apoptosis. Bcl‐2 prevents apoptosis by maintaining mitochondrial membrane integrity, whereas Bax, another member of the Bcl‐2 family, exhibits pro‐apoptotic effects. Caspases, a kind of cysteine protease, are important regulators of programmed cell death. Various apoptotic signaling pathways involve Caspase‐3. Apoptosis biomarkers include cleaved Caspase‐3, the active form of Caspase‐3, along with anti‐apoptotic Bcl‐2 and pro‐apoptotic Bax (Nagata 2018). In this study, the model group had significantly greater Bax/Bcl‐2 ratios and Cleaved Caspase‐3 levels than the control group, while RRFBs reduced the BLM‐induced alterations, particularly in the high‐dose groups. This demonstrated that RRFBs might inhibit apoptosis in alveolar epithelial cells, thereby mitigating PF.

An imbalance between the generation of reactive oxygen species (ROS), reactive nitrogen species (RNS), and antioxidant defenses results in oxidative stress, which damages tissue structure and causes cellular malfunction (Hosseinzadeh et al. 2018). It is commonly known that oxidative stress contributes to the PF process. Because oxidative stress causes damage to epithelial cells and encourages apoptosis, it can result in PF (Otoupalova et al. 2011). For instance, inflammatory cells in the lungs of patients with PF release larger quantities of oxidants compared with healthy individuals (Kinnula et al. 2005). Additionally, the production of ROS by mitochondria results in alveolar epithelial cells going through apoptosis in addition to cellular oxidative stress. To mitigate damage from oxidative stress, the body has a variety of antioxidant defense molecules, among which Nrf2 is one of the most crucial regulators. Upon stimulation, ROS and RNS modify the cysteine residues of Kelch‐like ECH‐associated protein 1 (Keap1), inhibiting the ubiquitination of Nrf2, promoting its phosphorylation, uncoupling the Nrf2‐Keap1 dimer, facilitating the translocation of Nrf2 to the nucleus, binding to the ARE, and initiating the release of SOD, HO‐1, NQO1, CAT, and other downstream antioxidant enzymes to maintain cellular redox homeostasis (Wang et al. 2022b). MDA is regarded as a critical sign of oxidative stress since it is a marker of lipid peroxidation, which causes irreversible tissue damage. Superoxide radicals are broken down into hydrogen peroxide and expelled from the body by SOD and GSH, two significant antioxidants and free radical scavengers (Lei et al. 2015). Exposure to BLM can result in DNA damage, increased ROS production, decreased T‐SOD, CAT, and GSH activity, and elevated MDA expression. In this study, lung tissues of the model group had much lower levels of GSH and SOD activity than the control group, but significantly greater levels of MDA. RRFBs intervention decreased MDA levels, enhanced GSH and SOD activity, and mitigated BLM‐induced oxidative stress. Patients with IPF have decreased Nrf2 expression in their fibroblasts and myofibroblasts (Han et al. 2021). HO‐1 and NQO1, key antioxidant enzymes downstream of Nrf2, regulate apoptosis by protecting against oxidative damage and contribute to cellular adaptation to oxidative stress (Loboda et al. 2016). It has been demonstrated that HO‐1 and NQO1 can exert protective effects in animal models of PF; for example, atractylenolide III inhibits BLM‐induced PF and oxidative stress via the Nrf2/NQO1/HO‐1 signaling pathway (Huai and Ding 2020). Immunofluorescence results visually demonstrated that Nrf2 positive fluorescence signals and nucleation into the nucleus were much lower in the model group. RRFBs facilitated the translocation of Nrf2 into the nucleus, leading to increased production of downstream antioxidant enzymes (HO‐1 and NQO1) and activated the Nrf2/HO‐1/NQO1 signaling pathway. RRFBs reduced oxidative stress damage caused by BLM in vivo, thereby mitigating lung fibrosis.

The gut microbiota, a complex micro‐ecosystem, is critical for maintaining health and preventing disease (Nie et al. 2018). Substantial evidence suggests that dietary therapy enhances immunity, modulates gut microbiota, and maintains intestinal homeostasis (García‐Montero et al. 2021). Gut microecological imbalances are associated with lung diseases, which can exacerbate the course of lung illness, compromise the integrity of the gut barrier, and trigger the systemic immune system (Wedgwood et al. 2020). Thus, it is crucial to preserve gut homeostasis in order to lessen pulmonary conditions like PF. 16S rDNA sequencing analysis indicated that BLM alters the intestinal microbial composition, increasing harmful bacteria, thereby promoting inflammatory responses and exacerbating PF. The current study discovered that the relative abundance of *Proteobacteria* was markedly higher, and the relative abundance of *Firmicutes* was lower in the model group at the phylum level, and these BLM‐induced changes were attenuated by RRFBs. At the genus level, the relative abundance of pathogenic bacteria *Proteus* and *Escherichia‐Shigella* was higher, while the relative abundance of probiotics *Ileibacterium* and *Dubosiella* was markedly lower in the model group; however, the relative abundance of *Ileibacterium* and *Dubosiella* increased notably after treatment with RRFBs. *Proteus* and *Escherichia‐Shigella* are major genera in *Proteobacteria*. Patients experiencing exacerbations of chronic obstructive pulmonary disease have high levels of *Escherichia‐Shigella* in the intestines (Gupta et al. 2021). *Ileibacterium* and *Dubosiella*, belonging to *Firmicutes*, are probiotic bacteria crucial for gut health in humans. Probiotics help regulate elevated levels of opportunistic pathogens in gut microbiota, restoring balance and enhancing intestinal mucosal immunity. *Ileibacterium* ameliorates intestinal inflammation and enhances intestinal permeability; however, its abundance is reduced in mice with DSS‐induced colitis (Shao et al. 2022). *Dubosiella*, acting as an antioxidant, regulates metabolism, enhances intestinal immunity, and protects against inflammatory diseases. Its abundance was notably reduced in radiation‐induced experimental PF (Li et al. 2020), in line with the findings of our current research. Furthermore, *Dubosiella* has demonstrated the ability to lower oxidative stress in mice and exhibits potential anti‐aging properties (Liu et al. 2023b). According to these findings, RRFBs might ameliorate BLM‐induced PF by affecting the gut microbiota.

Both oxidative stress and gut microbiota influence PF. Therefore, we further explored functional correlations between gut microbiota and oxidative stress, revealing that MDA was markedly negatively correlated with the probiotic bacteria *Ileibacterium* and *Dubosiella* and markedly positively correlated with pathogenic bacteria including *Enterococcus*, *Staphylococcus*, *Escherichia‐Shigella*, and *Bacteroides*. These findings establish a link between the severity of oxidative stress and the abundance of probiotics and pathogenic bacteria. Antioxidants SOD and GSH both had significant positive correlations with the probiotic *Ileibacterium* and had a substantial negative correlation with the pathogenic *Enterococcus*. In addition, SOD showed a substantial negative correlation with *Staphylococcus* while a significant positive correlation with *Dubosiella*, *Turicibacter*, and *Clostridium_sensu_stricto_1*. GSH was significantly negatively correlated with *Bacteroides*. *Turicibacter* has been noted to alleviate inflammation (Liu et al. 2023a). *Clostridium_sensu_stricto_1*, a member of the phylum *Firmicutes*, is increased in a rat PF model treated with pirfenidone (Gao et al. 2023). A heatmap revealed that the associations between SOD, GSH, and the gut microbiota were similar and were generally the opposite of those for MDA, consistent with the results showing that RRFBs inhibit oxidative stress in BLM‐induced PF. Overall, gut microbiota and oxidative stress were closely correlated; to be specific, the inhibition of oxidative stress was positively associated with probiotics and negatively associated with pathogenic bacteria.

Overall, both RRFBs effectively ameliorated BLM‐induced PF in mice by reducing inflammatory responses, inhibiting the EMT, oxidative stress, and apoptosis, and alleviating gut microbial dysbiosis.

## 5 Conclusions

In the present study, we found that two RRFBs were rich in organic acids, polyphenols, flavonoids, and other bioactive components. JB3 had more active components and stronger antioxidant activity than those of JB1, which may explain its better anti‐PF effect in vivo. RRFBs ameliorated BLM‐induced PF, with better effects in the high‐dose groups (8 mL/kg) than in the low‐dose groups (4 mL/kg). With respect to underlying mechanisms, RRFBs ameliorated inflammatory responses, EMT, oxidative stress, and apoptosis by activating the Nrf2/HO‐1/NQO1 signaling pathway. They also attenuated alterations in the gut microbiota in PF; in particular, RRFBs decreased the relative abundance of the pathogenic bacterium *Proteus* while increasing the relative abundance of probiotics *Ileibacterium* and *Dubosiella*. This research may serve as a reference for the development of functional RRT foods and for advancing research on the treatment of PF.

## Author Contributions

**Heting Zhou:** conceptualization (equal), visualization (equal), writing – original draft (equal). **Xinyue Zheng:** methodology (equal). **Shaolin Huang:** investigation (equal), methodology (equal). **Xiaomeng Wang:** investigation (equal), visualization (equal). **Ting Zhou:** methodology (equal). **Shuwen Zhang:** visualization (equal). **Yihan Ling:** methodology (equal). **Wenxi Wang:** methodology (equal). **Xingjie Li:** formal analysis (equal). **Shouqian Li:** funding acquisition (equal). **Yongmei Xie:** supervision (equal). **Wenya Yin:** conceptualization (equal), funding acquisition (equal), supervision (equal).

## Conflicts of Interest

The authors declare no conflicts of interest.

## Supporting information


Data S1.

## Data Availability

The data that support the findings of this study are available on request from the corresponding author.
