# Supplementary material for: Rosa roxburghii Fermentation Broths Attenuate Bleomycin‐Induced Pulmonary Fibrosis by Activating the Nrf2/HO‐1/NQO1 Signaling Pathway and Modulating Gut Microbiota
Source: Food Sci Nutr. 2025 Mar 19;13(3):e70105. doi: 10.1002/fsn3.70105 (PMC11923242; doi:10.1002/fsn3.70105)
Supplement: Supplementary file 1 — Data S1. [file FSN3-13-e70105-s001.docx]

**
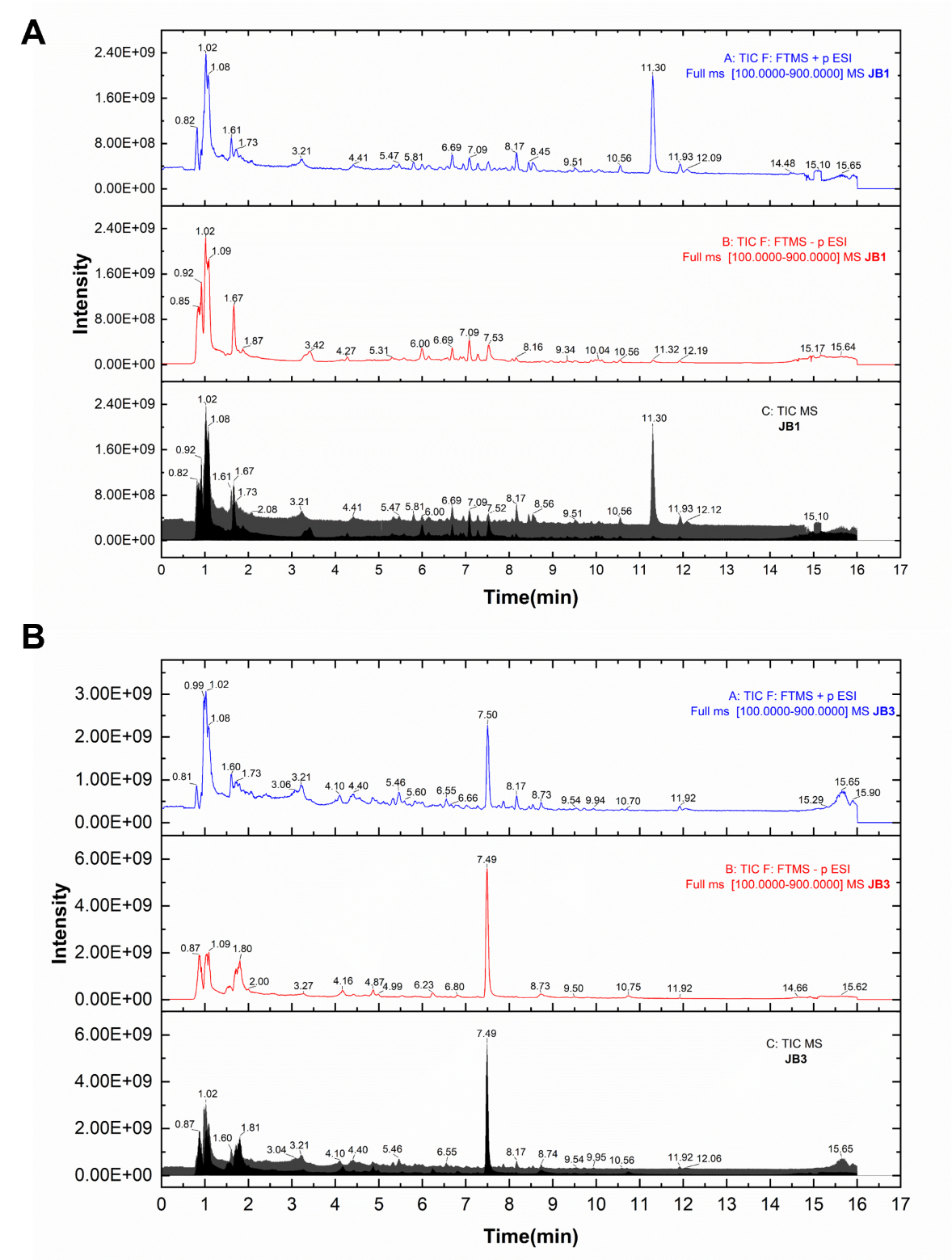
**

**Supplementary Figure. S1 The total ion chromatogram (TIC) in RRFBs.** (A) JB1. (B) JB3. RRFBs was scanned in FTMS + p ESI, FTMS - p ESI, and full scan mode with a scan range of 100–900 m/z using HPLC-Q-Exactive Orbitrap-MS.

**Supplementary Table S1 Possible components in RRFBs.**

| **No.** | **Name** | **Formula** | **Molecular Weight** | **RT （min）** | **Reference Ion** | **m/z** | **Ratio: (JB3 / JB1)** | **Classification** |
| --- | --- | --- | --- | --- | --- | --- | --- | --- |
| 1 | Citric acid | C6 H8 O7 | 192.0268 | 0.857 | [M-H]^-^1 | 191.0195 | 0.3307 | Organic acids |
| 2 | DL-Malic acid | C4 H6 O5 | 134.0214 | 0.859 | [M-H]^-^1 | 133.0141 | 1.7036 | Organic acids |
| 3 | DL-Lysine | C6 H14 N2 O2 | 146.1055 | 0.862 | [M+H-NH3]^+^1 | 130.0862 | 2.2472 | Amino acids |
| 4 | Fumaric acid | C4 H4 O4 | 116.0109 | 0.862 | [M-H]^-^1 | 115.0036 | 1.7857 | Organic acids |
| 5 | L-Threonic acid | C4 H8 O5 | 136.0369 | 0.912 | [M-H]^-^1 | 135.0298 | 0.3298 | Organic acids |
| 6 | L-Histidine | C6 H9 N3 O2 | 155.0695 | 0.938 | [M+H]^+^1 | 156.0767 | 2.4938 | Amino acids |
| 7 | DL-Arginine | C6 H14 N4 O2 | 174.1116 | 0.939 | [M+H]^+^1 | 175.1189 | 0.6803 | Amino acids |
| 8 | Asparagine | C4 H8 N2 O3 | 132.0535 | 0.956 | [M+H]^+^1 | 133.0607 | 1.2953 | Amino acids |
| 9 | Choline | C5 H13 N O | 103.0997 | 0.967 | [M+H]^+^1 | 104.1069 | 1.1765 | Vitamins |
| 10 | L-Aspartic acid | C4 H7 N O4 | 133.0375 | 0.974 | [M+H]^+^1 | 134.0448 | 0.8673 | Amino acids |
| 11 | L-Serine | C3 H7 N O3 | 105.0426 | 0.974 | [M-H]^-^1 | 104.0353 | 2.2779 | Amino acids |
| 12 | D-(-)-Mannitol | C6 H14 O6 | 182.0788 | 0.999 | [M-H]^-^1 | 181.0715 | 0.2854 | Sugar alcohols |
| 13 | L-Glutamic acid | C5 H9 N O4 | 147.0531 | 1.001 | [M+H]^+^1 | 148.0604 | 0.4500 | Amino acids |
| 14 | Gallic acid | C7 H6 O5 | 170.0214 | 1.001 | [M-H]^-^1 | 169.0141 | 2.6525 | Polyphenols |
| 15 | Betaine | C5 H11 N O2 | 117.0789 | 1.015 | [M+H]^+^1 | 118.0862 | 1.3333 | Alkaloids |
| 16 | 4-Oxoproline | C5 H7 N O3 | 129.0425 | 1.018 | [M-H]^-^1 | 128.0352 | 1.8657 | Organic acids |
| 17 | D-(+)-Mannose | C6 H12 O6 | 180.0632 | 1.039 | [M-H]^-^1 | 179.0559 | 0.8439 | Saccharides |
| 18 | D-(-)-Quinic acid | C7 H12 O6 | 192.0631 | 1.046 | [M-H]^-^1 | 191.0559 | 0.4179 | Organic acids |
| 19 | α,α-Trehalose | C12 H22 O11 | 342.1158 | 1.051 | [M-H]^-^1 | 341.1085 | 1.0571 | Saccharides |
| 20 | D-(-)-Ribose | C5 H10 O5 | 150.0526 | 1.056 | [M-H]^-^1 | 149.0454 | 0.5507 | Saccharides |
| 21 | Trigonelline | C7 H7 N O2 | 137.0476 | 1.058 | [M+H]^+^1 | 138.0549 | 5.9524 | Alkaloids |
| 22 | Proline | C5 H9 N O2 | 115.0633 | 1.072 | [M+H]^+^1 | 116.0705 | 1.0352 | Amino acids |
| 23 | Gluconic acid | C6 H12 O7 | 196.0581 | 1.08 | [M-H]^-^1 | 195.0508 | 0.3525 | Organic acids |
| 24 | DL-Stachydrine | C7 H13 N O2 | 143.0946 | 1.094 | [M+H]^+^1 | 144.1018 | 0.6693 | Alkaloids |
| 25 | Adenine | C5 H5 N5 | 135.0545 | 1.097 | [M+H]^+^1 | 136.0617 | 0.6098 | Vitamins |
| 26 | Valine | C5 H11 N O2 | 117.079 | 1.118 | [M+H]^+^1 | 118.0862 | 3.3445 | Amino acids |
| 27 | L-(-)-Arabitol | C5 H12 O5 | 152.0683 | 1.128 | [M-H]^-^1 | 151.061 | 11.1111 | Sugar alcohols |
| 28 | Succinic acid | C4 H6 O4 | 118.0266 | 1.424 | [M-H]^-^1 | 117.0193 | 0.5208 | Organic acids |
| 29 | Methionine | C5 H11 N O2 S | 149.051 | 1.652 | [M+H]^+^1 | 150.0583 | 3.7736 | Amino acids |
| 30 | L-Pyroglutamic acid | C5 H7 N O3 | 129.0426 | 1.868 | [M+H]^+^1 | 130.0499 | 2.8571 | Organic acids |
| 31 | Isoleucine | C6 H13 N O2 | 131.0946 | 2.236 | [M+H]^+^1 | 132.1019 | 5.3763 | Amino acids |
| 32 | Protocatechuic acid | C7 H6 O4 | 154.0265 | 2.414 | [M-H] ^-^1 | 153.0192 | 0.5023 | Polyphenols |
| 33 | Leucine | C6 H13 N O2 | 131.0946 | 2.444 | [M+H]^+^1 | 132.1019 | 6.6225 | Amino acids |
| 34 | Vanillic acid | C8 H8 O4 | 168.0421 | 2.464 | [M-H] ^-^1 | 167.0348 | 0.5650 | Polyphenols |
| 35 | L-Phenylalanine | C9 H11 N O2 | 165.0789 | 3.685 | [M-H] ^-^1 | 164.0716 | 3.9683 | Amino acids |
| 36 | Benzoic acid | C7 H6 O2 | 122.0367 | 4.048 | [M-H] ^-^1 | 121.0294 | 0.0673 | Organic acids |
| 37 | 4-Pyridoxic acid | C8 H9 N O4 | 183.0532 | 4.316 | [M+H]^+^1 | 184.0605 | 30.3030 | Vitamins |
| 38 | L-Phenylalanine | C9 H11 N O2 | 165.0789 | 4.415 | [M+H]^+^1 | 166.0862 | 3.6630 | Amino acids |
| 39 | Tyrosol | C8 H10 O2 | 138.0679 | 4.776 | [M-H+HAc]^-^1 | 197.0817 | 0.0214 | Polyphenols |
| 40 | 8-Hydroxyquinoline | C9 H7 N O | 145.0527 | 4.777 | [M+H]^+^1 | 146.06 | 0.0519 | Phenols |
| 41 | Theobromine | C7 H8 N4 O2 | 180.0648 | 4.988 | [M+H]^+^1 | 181.0721 | 9.4340 | Alkaloids |
| 42 | D-(+)-Tryptophan | C11 H12 N2 O2 | 204.0897 | 5.041 | [M-H] ^-^1 | 203.0824 | 6.8493 | Amino acids |
| 43 | 1,6-Bis-O-(3,4,5-trihydroxybenzoyl)hexopyranose | C20 H20 O14 | 484.0852 | 5.051 | [M-H] ^-^1 | 483.0779 | 43.4783 | Glycosides |
| 44 | Prolylleucine | C11 H20 N2 O3 | 228.1475 | 5.056 | [M+H]^+^1 | 229.1547 | 5.6497 | Amino acids |
| 45 | Chlorogenic acid | C16 H18 O9 | 354.0948 | 5.118 | [M-H] ^-^1 | 353.0876 | 5.4054 | Polyphenols |
| 46 | Azelaic acid | C9 H16 O4 | 188.1047 | 5.198 | [M-H] ^-^1 | 187.0975 | 2.1739 | Organic acids |
| 47 | Luteolin-3',7-Diglucoside | C27 H30 O16 | 610.1534 | 5.207 | [M+H]^+^1 | 611.1606 | 0.8787 | Glycosides |
| 48 | Leucylproline | C11 H20 N2 O3 | 228.1474 | 5.375 | [M+H]^+^1 | 229.1547 | 4.5662 | Amino acids |
| 49 | trans-3-Indoleacrylic acid | C11 H9 N O2 | 187.0633 | 5.587 | [M+H]^+^1 | 188.0706 | 0.1255 | Organic acids |
| 50 | 2,4-Quinolinediol | C9 H7 N O2 | 161.0477 | 5.83 | [M+H]^+^1 | 162.055 | 14.2857 | Polyphenols |
| 51 | Catechol | C6 H6 O2 | 110.0367 | 6.034 | [M-H] ^-^1 | 109.0294 | 0.2262 | Phenols |
| 52 | Cnidioside A | C17 H20 O9 | 368.1104 | 6.058 | [M-H] ^-^1 | 367.1032 | 2.3041 | Glycosides |
| 53 | Catechin | C15 H14 O6 | 290.0788 | 6.144 | [M-H] ^-^1 | 289.0716 | 0.0018 | Flavonoids |
| 54 | Salicylic acid | C7 H6 O3 | 138.0316 | 6.148 | [M+H]^+^1 | 139.0389 | 0.0804 | Organic acids |
| 55 | Genistin | C21 H20 O10 | 432.1056 | 6.244 | [M+H]^+^1 | 433.1129 | 0.1963 | Glycosides |
| 56 | 2-(2-amino-3-methylbutanamido)-3-phenylpropanoic acid | C14 H20 N2 O3 | 264.1475 | 6.386 | [M+H]^+^1 | 265.1548 | 4.6512 | Amino acids |
| 57 | Epicatechin | C15 H14 O6 | 290.079 | 6.643 | [M+H]^+^1 | 291.0863 | 0.1742 | Flavonoids |
| 58 | Orientin | C21 H20 O11 | 448.1007 | 6.886 | [M+H]^+^1 | 449.1079 | 2.9762 | Flavonoids |
| 59 | Cynaroside | C21 H20 O11 | 448.1003 | 6.998 | [M-H] ^-^1 | 447.093 | 0.5945 | Glycosides |
| 60 | 4-Anisic acid | C8 H8 O3 | 152.0472 | 7.116 | [M-H] ^-^1 | 151.0399 | 0.1904 | Organic acids |
| 61 | Salicylic acid | C7 H6 O3 | 138.0316 | 7.317 | [M-H] ^-^1 | 137.0243 | 0.3914 | Organic acids |
| 62 | Lariciresinol 4-O-glucoside | C26 H34 O11 | 522.2101 | 7.364 | [M-H] ^-^1 | 521.2029 | 1.5198 | Flavonoids |
| 63 | Abscisic acid | C15 H20 O4 | 264.136 | 7.372 | [M-H] ^-^1 | 263.1287 | 1.2136 | Organic acids |
| 64 | Vitexin | C21 H20 O10 | 432.1057 | 7.4 | [M+H]^+^1 | 433.113 | 1.1062 | Flavonoids |
| 65 | Maltol | C6 H6 O3 | 126.0317 | 7.495 | [M+H]^+^1 | 127.0389 | 12.1951 | Phenols |
| 66 | Vanillin | C8 H8 O3 | 152.0471 | 7.519 | [M+FA-H]^-^1 | 197.0453 | 12.1951 | Aldehydes |
| 67 | Eriodictyol | C15 H12 O6 | 288.0634 | 7.526 | [M+H]^+^1 | 289.0707 | 0.9363 | Flavonoids |
| 68 | 7-Hydroxycoumarine | C9 H6 O3 | 162.0317 | 7.85 | [M+H]^+^1 | 163.0389 | 0.6671 | Polyphenols |
| 69 | Taxifolin | C15 H12 O7 | 304.0584 | 7.907 | [M+H]^+^1 | 305.0657 | 1.2210 | Flavonoids |
| 70 | Neodiosmin | C28 H32 O15 | 608.1739 | 7.971 | [M+H]^+^1 | 609.1812 | 1.3423 | Glycosides |
| 71 | Hesperidin | C28 H34 O15 | 610.1895 | 8.067 | [M+H]^+^1 | 611.1968 | 0.7008 | Glycosides |
| 72 | Naringenin | C15 H12 O5 | 272.0684 | 8.142 | [M+H]^+^1 | 273.0756 | 2.9499 | Flavonoids |
| 73 | Afzelin | C21 H20 O10 | 432.1054 | 8.49 | [M-H]^-^1 | 431.0981 | 15.3846 | Flavonoids |
| 74 | Myricetin | C15 H10 O8 | 318.0376 | 8.502 | [M+H]^+^1 | 319.0449 | 52.6316 | Flavonoids |
| 75 | Myricetin | C15 H10 O8 | 318.0374 | 8.52 | [M-H]^-^1 | 317.0301 | 8.7719 | Flavonoids |
| 76 | 4-Anisic acid | C8 H8 O3 | 152.0473 | 8.896 | [M+H]^+^1 | 153.0546 | 0.1294 | Organic acids |
| 77 | Corchorifatty acid F | C18 H32 O5 | 328.2247 | 9.016 | [M-H]^-^1 | 327.2175 | 0.1503 | Organic acids |
| 78 | Eriodictyol | C15 H12 O6 | 288.0632 | 9.339 | [M-H]^-^1 | 287.0559 | 0.5038 | Flavonoids |
| 79 | Kaempferol | C15 H10 O6 | 286.0477 | 9.436 | [M+H]^+^1 | 287.055 | 1.2438 | Flavonoids |
| 80 | Quercetin | C15 H10 O7 | 302.0426 | 9.487 | [M+H]^+^1 | 303.0499 | 5.1546 | Flavonoids |
| 81 | Quercetin | C15 H10 O7 | 302.0425 | 9.512 | [M-H]^-^1 | 301.0352 | 3.8023 | Flavonoids |
| 82 | Naringeninchalcone | C15 H12 O5 | 272.0685 | 10.256 | [M+H]^+^1 | 273.0758 | 1.0537 | Flavonoids |
| 83 | Luteolin | C15 H10 O6 | 286.0478 | 10.404 | [M+H]^+^1 | 287.0551 | 1.6750 | Flavonoids |
| 84 | Luteolin | C15 H10 O6 | 286.0475 | 10.411 | [M-H]^-^1 | 285.0403 | 1.4663 | Flavonoids |
| 85 | Isorhamnetin | C16 H12 O7 | 316.0581 | 10.561 | [M-H]^-^1 | 315.0509 | 0.9251 | Flavonoids |
| 86 | Hesperetin | C16 H14 O6 | 302.079 | 10.563 | [M+H]^+^1 | 303.0863 | 1.2484 | Flavonoids |
| 87 | Isorhamnetin | C16 H12 O7 | 316.0582 | 10.576 | [M+H]^+^1 | 317.0654 | 1.2151 | Flavonoids |
| 88 | Linolenic Acid | C18 H30 O2 | 278.2243 | 14.792 | [M-H]^-^1 | 277.217 | 20.0000 | Organic acids |
| 89 | Stearic acid | C18 H36 O2 | 284.2713 | 14.956 | [M-H]^-^1 | 283.264 | 1.1468 | Organic acids |
| 90 | Oleic acid | C18 H34 O2 | 282.2556 | 15.503 | [M-H]^-^1 | 281.2483 | 1.1574 | Organic acids |
| 91 | Erucic acid | C22 H42 O2 | 338.3183 | 15.899 | [M-H]^-^1 | 337.311 | 35.7143 | Organic acids |

**Supplementary Table S2 Sequences of the primers for real-time qPCR analysis.**

| **Gene (species)** | **Sequences** | |
| --- | --- | --- |
| GAPDH (Mouse) | Forward | 5’-GGTTGTCTCCTGCGACTTCA-3’ |
|  | Reverse | 5’-TGGTCCACCCTTTCTTACTCC-3’ |
| Collagen I (Mouse) | Forward | 5’-TGTTCAGCTTTGTGGACCTC-3’ |
|  | Reverse | 5’-GGTTTCCACGTCTCACCATT-3’ |
| Vimentin (Mouse) | Forward | 5’-CGTGCGGCTGCTTCAAGACTC-3’ |
|  | Reverse | 5’-CTTCTCGTTGGTGCGGGTGTTC-3’ |
| E-cadherin (Mouse) | Forward | 5’-GGGCTGGACCGAGAGAGTTACC-3’ |
|  | Reverse | 5’-CGTGCTTGGGTTGAAGACAGGAG-3’ |
| IL-1β (Mouse) | Forward | 5’-CACTACAGGCTCCGAGATGAACAAC-3’ |
|  | Reverse | 5’-TGTCGTTGCTTGGTTCTCCTTGTAC-3’ |
| IL-6 (Mouse) | Forward | 5’-CTTCTTGGGACTGATGCTGGTGAC-3’ |
|  | Reverse | 5’-TCTGTTGGGAGTGGTATCCTCTGTG-3’ |
| TNF-α (Mouse) | Forward | 5’-CGCTCTTCTGTCTACTGAACTTCGG-3’ |
|  | Reverse | 5’-GTGGTTTGTGAGTGTGAGGGTCTG-3’ |
| IL-10 (Mouse) | Forward | 5’-GCTCTTACTGACTGGCATGAG-3’ |
|  | Reverse | 5’-CGCAGCTCTAGGAGCATGTG-3’ |
| SOD1 (Mouse) | Forward | 5’-TAACTGAAGGCCAGCATGGGT-3’ |
|  | Reverse | 5’-GGTCTCCAACATGCCTCTCTTC-3’ |
| GPX2 (Mouse) | Forward | 5’-GAACGAGGAGATCCTGAACAGC-3’ |
|  | Reverse | 5’-GGTAGGGCAGCTTGTCTTTCAG-3’ |
| CAT (Mouse) | Forward | 5’-TTGTTCAGTGACCGAGGGATT-3’ |
|  | Reverse | 5’-TTCCTGAGCAAGCCTTCCTG-3’ |
| Nrf2 (Mouse) | Forward | 5’-ACTTCGGTGATTCTGTTAG-3’ |
|  | Reverse | 5’-GGTAGGGCAGCTTGTCTTTCAG-3’ |
| HO-1 (Mouse) | Forward | 5’-ACCGCCTTCCTGCTCAACATTG-3’ |
|  | Reverse | 5’-CTCTGACGAAGTGACGCCATCTG-3’ |
| NQO1 (Mouse) | Forward | 5’-CATTGCAGTGGTTTGGGGTG-3’ |
|  | Reverse | 5’-TCTGGAAAGGACCGTTGTCG-3’ |
